# Unexpected cause of pancytopenia in a teenager with Noonan syndrome

**DOI:** 10.1002/jha2.225

**Published:** 2021-05-21

**Authors:** Salvatore Perrone, Natalia Cenfra, Federica Viola, Anna Carraro, Raffaella Marocco, Miriam Lichtner, Giuseppe Cimino

**Affiliations:** ^1^ Hematology Polo Universitario Pontino S.M. Goretti Hospital Latina Italy; ^2^ Department of Medical Oncology “Sapienza University of Rome” Medical and Surgical Sciences and Biotechnology Rome Italy; ^3^ Department of Public Health and Infectious Diseases “Sapienza University” S.M. Goretti Hospital Latina Italy

**Keywords:** bleeding disorders, Leishmania, Noonan syndrome, thrombocytopenia

Noonan syndrome (NS) is an autosomal dominant genetic condition characterized by hyperactivation of the *RAS/MAPK* signaling pathway, caused by mutations in various genes, the most frequent being *PTPN11*, mutated in about 50% of cases. Typical features of NS include characteristic facial features, short stature, congenital heart defect, skeletal and thoracic anomalies, developmental delay, and bleeding problems, the latter resulting from defects in coagulation or platelets or both; these are seen in 30–72% of patients. Cases of hematological neoplasms have also been reported in patients with NS, particularly in the pediatric age; the most frequent are juvenile myelomonocytic leukemia, acute myelogenous leukemia, and B‐cell acute lymphoblastic leukemia.

A 20‐year‐old young man with NS went to the hospital due to the onset of fever, bleeding gums and epistaxis. Past medical history included surgery for hydrocele at the age of 2 and the physical examination revealed a palpable spleen. Blood chemistry tests showed pancytopenia (Haemoglobin [Hb] 94 g/l; white blood cells [WBC] 1.67 × 10^9^/l; neutrophils [N] 0.6 × 10^9^/l; lymphocytes [Ly] 0.71 × 10^9^/l; platelets [PLT] 11 × 10^9^/l), normal coagulation, C‐reactive protein 4.63 mg/dl and hypergammaglobulinemia. Ultrasound of the abdomen showed hepatomegaly and voluminous splenomegaly (Figure 1A) that reached the left iliac fossa. Given his symptoms and history of NS, a suspicion of acute leukemia raised and we performed a hematological evaluation with bone marrow aspiration. Conversely, marrow cytology revealed reactive plasma cells and the presence of intra and extracellular amastigotes (Figure 1B), very suspicious for leishmaniasis, that is endemic in the Mediterranean basin. Indeed, serology for Leishmania was positive (IgG > 1:320) and bone marrow sample, analyzed by polymerase chain reaction (PCR), was positive for *L. infantum*, establishing a diagnosis of visceral leishmaniasis (VL). VL is caused by a primary infection with Leishmania parasites transmitted by the bite of infected female phlebotomine sandflies. Clinical features of the typical forms are fever, weight loss, hepatosplenomegaly and pancytopenia. The invasion of the bone marrow by the parasite causes anemia, thrombocytopenia––worsened by hypersplenism––which increases the risk of bleeding, and leukopenia which predisposes to further infections. The patient was admitted to the infectious disease ward and received liposomal amphotericin B on days 1–5, 14, and 21, was supported with platelets transfusion and symptoms improved rapidly. Six months later, splenomegaly (15 cm) and anemia improved, while thrombocytopenia remained unchanged (Hb 120 g/l; WBC 3.73 × 10^9^/l; neutrophils 1.84 × 10^9^/l; Ly 1.50 × 10^9^/l PLT 30 × 10^9^/l), PCR for Leishmania was negative, karyotype and flow‐cytometry were negative for myelodysplastic syndrome. Frequently bleeding disorders and thrombocytopenia are reported in patients with NS. Even if ears and urinary infections are reported in NS––related with some extent of hypogammaglobulinemia––this is a rare report of VL in these patients, mimicking acute leukemia at presentation.

**FIGURE 1:**
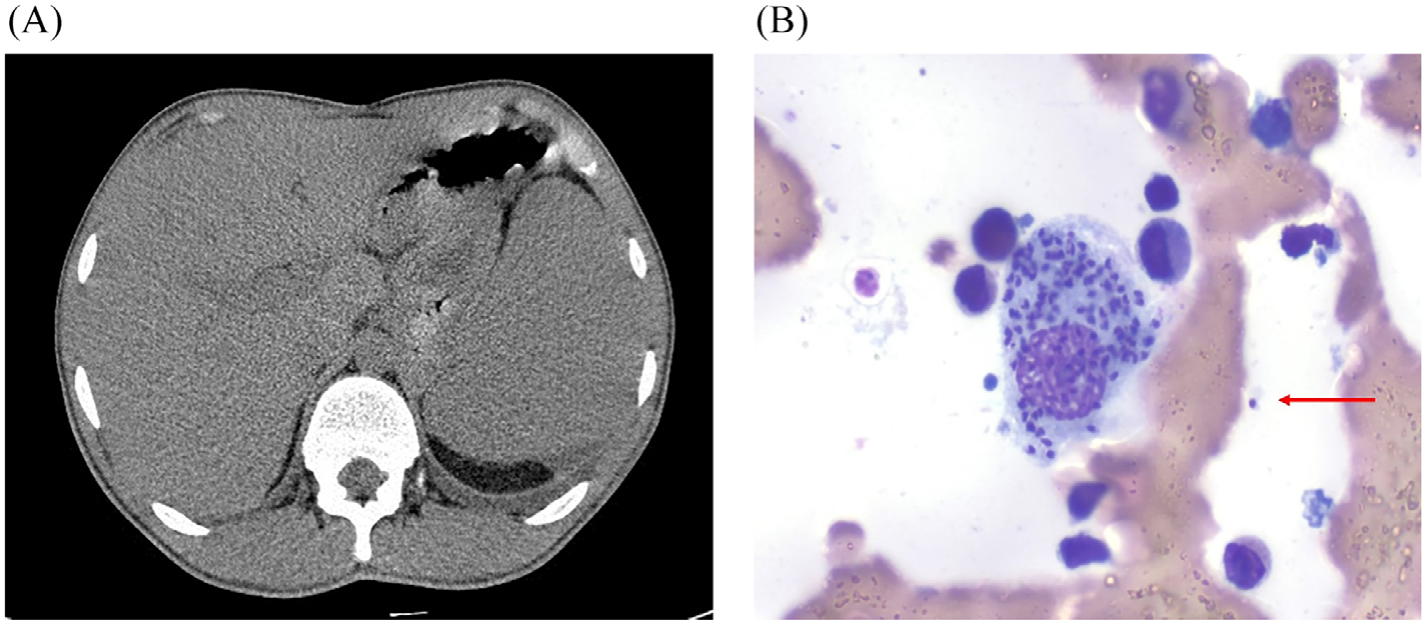
(A): CT scan showing an enlarged spleen. (B): Bone marrow smear showing several amastigotes engulfed by a macrophage. A free amastigote can also be seen (red arrow)

## CONFLICT OF INTEREST

The authors declare no conflict of interest. This work was unfunded.

